# Continuous carbon dioxide monitoring in the exhaled breath of mechanically ventilated rats

**DOI:** 10.1113/EP093058

**Published:** 2025-09-14

**Authors:** Christin Wenzel, Silke Borgmann, Bernd Flamm, Lea Kuhn, Stefan Schumann, Johannes Schmidt, Johannes Spaeth, Sashko Spassov

**Affiliations:** ^1^ Department of Anesthesiology and Critical Care Medical Center – University of Freiburg, Faculty of Medicine, University of Freiburg Freiburg Germany

**Keywords:** carbon dioxide, CO_2_ partial pressure, end‐tidal, mechanical ventilation, monitoring, respiratory efficiency, small animals

## Abstract

In small animals, carbon dioxide monitoring is either limited by the need to take blood samples for gas analysis, or it interferes with respiratory efficiency and lung mechanics analysis. We introduced a novel approach for continuous monitoring of CO_2_ in the expiratory limb of the breathing circuit. The relevance of the method is assessed by CO_2_ measurements at different respiratory settings. Rats were ventilated with a tidal volume (*V*_T_) of 8 mL kg^−1^. Respiratory rates were adjusted to achieve arterial CO_2_ partial pressure ($P_{\mathrm{aC}O_{2}}$) between 35 and 45 mmHg. We measured partial pressure of CO_2_ in the expiratory limb of the breathing circuit (exCO_2_). exCO_2_ values were compared to $P_{\mathrm{aC}O_{2}}$ from blood gas analysis. The agreement between the two measurements was assessed by correlation and Bland–Altman analysis. The validity of the novel approach was established through additional experimental runs with *V*_T_ of 7 or 6 mL kg^−1^, where the respiratory rate was set in accordance to exCO_2_. Measurements of exCO_2_ reflected $P_{\mathrm{aC}O_{2}}$ with high correlation (*R* ^2 ^= 0.8658). Bland–Altman analysis showed high agreement between the two measurements. Respiratory rate setting guided by exCO_2_ (39 ± 2 or 40 ± 2 mmHg during ventilation with *V*_T_ of 7 or 6 mL kg^−1^, respectively) was appropriate to maintain $P_{\mathrm{aC}O_{2}}$ (41 ± 2 mmHg for both *V*_T_). Measurement of exCO_2_ provides a robust estimate of $P_{\mathrm{aC}O_{2}}$ in anaesthetized small animals during mechanical ventilation. Continuous monitoring of CO_2_ in the exhaled breath could be used to guide mechanical ventilation settings.

## Abstract (highlights)

- **What is** **the central question of this study?**
  Is continuous monitoring of CO_2_ in the expiratory limb of the breathing circuit in small animals under mechanical ventilation feasible to guide mechanical ventilation settings?
- **What is the main finding and its importance?**
  Continuous monitoring of exhaled CO_2_ is a valid proxy for $P_{\mathrm{aC}O_{2}}$ and allows for its maintenance in the physiological range during mechanical ventilation of healthy rats. Its application reduces the amount of blood required for blood gas analysis, without compromising respiratory efficiency or the quality of respiratory mechanics data.

## 1 INTRODUCTION

Small animal models are indispensable in preclinical research. Animal studies often include surgical interventions. In this regard, mechanical ventilation is essential for maintaining blood gas homeostasis in anaesthetized animals. Its efficiency is crucial for further testing and analysis since compromised blood gas balance substantially interferes with experimental conditions and may alter study results. For that reason, monitoring and maintaining blood gas balance is essential for ensuring quality, relevance and reproducibility of animal experiments (Wenzel et al., 2021). The effective use of mechanical ventilation relies on precise monitoring of the CO_2_ levels (Schmidt, 2020; Shannon, 1989). Measurement of CO_2_ based on blood gas analysis (BGA) is considered the gold standard. However, the use of BGA in preclinical research can be limited. In small animals in particular, the blood volume is low and sampling is challenging and may require surgical interventions, which may not be compatible with the study design or even compromise study results. Therefore, non‐invasive measurements, that is, capnometry, are available for monitoring respiratory efficiency. CO_2_ partial pressure measured at the end of expiration (end‐tidal CO_2_; $P_{\mathrm{ETC}O_{2}}$) approximates the arterial CO_2_ levels and is suitable for monitoring CO_2_ exchange dynamics. $P_{\mathrm{ETC}O_{2}}$ monitoring is configured as either mainstream or sidestream. In mainstream, the CO_2_ cuvette is placed between the Y‐piece connector and the endotracheal tube, which increases instrumental death space. Dead space is the air volume that does not contribute to gas exchange within the lungs. Due to the small *V*_T_ and high respiratory rates, typical for ventilation of small animals, the additional dead space reduces the respiratory efficiency to a higher degree than in large animals or humans. Dead space can be avoided by using sidestream $P_{\mathrm{ETC}O_{2}}$ monitoring, where the CO_2_ cuvette is separate from the airway, and exhaled gas is diverted through a sampling tube. However, the air sampled during sidestream CO_2_ measurement interferes with flow and pressure measurements and hinders proper analysis of the respiratory mechanics. To avoid these limitations, we have introduced and validated a continuous monitoring of exhaled CO_2_ partial pressure (exCO_2_) in the expiratory limb of the breathing circuit of ventilated small animals.

## 2 METHODS

### 2.1 Ethical approval

Animal experiments were approved by the local Animal Welfare Committee (Regierungspräsidium Freiburg; Approval number G23/43) and conducted in compliance with the German law and the European directive 2010/63/EU animal care and the ARRIVE guidelines for reporting animal research (Percie du Sert et al., 2020). Animals were kept 7–14 days in a holding facility prior the experiments for adaptation. Animal housing followed a 12/12 h day–night cycle. Animals had free access to water and food until the experiments.

### 2.2 Measurement of exCO_2_

**FIGURE 1:**
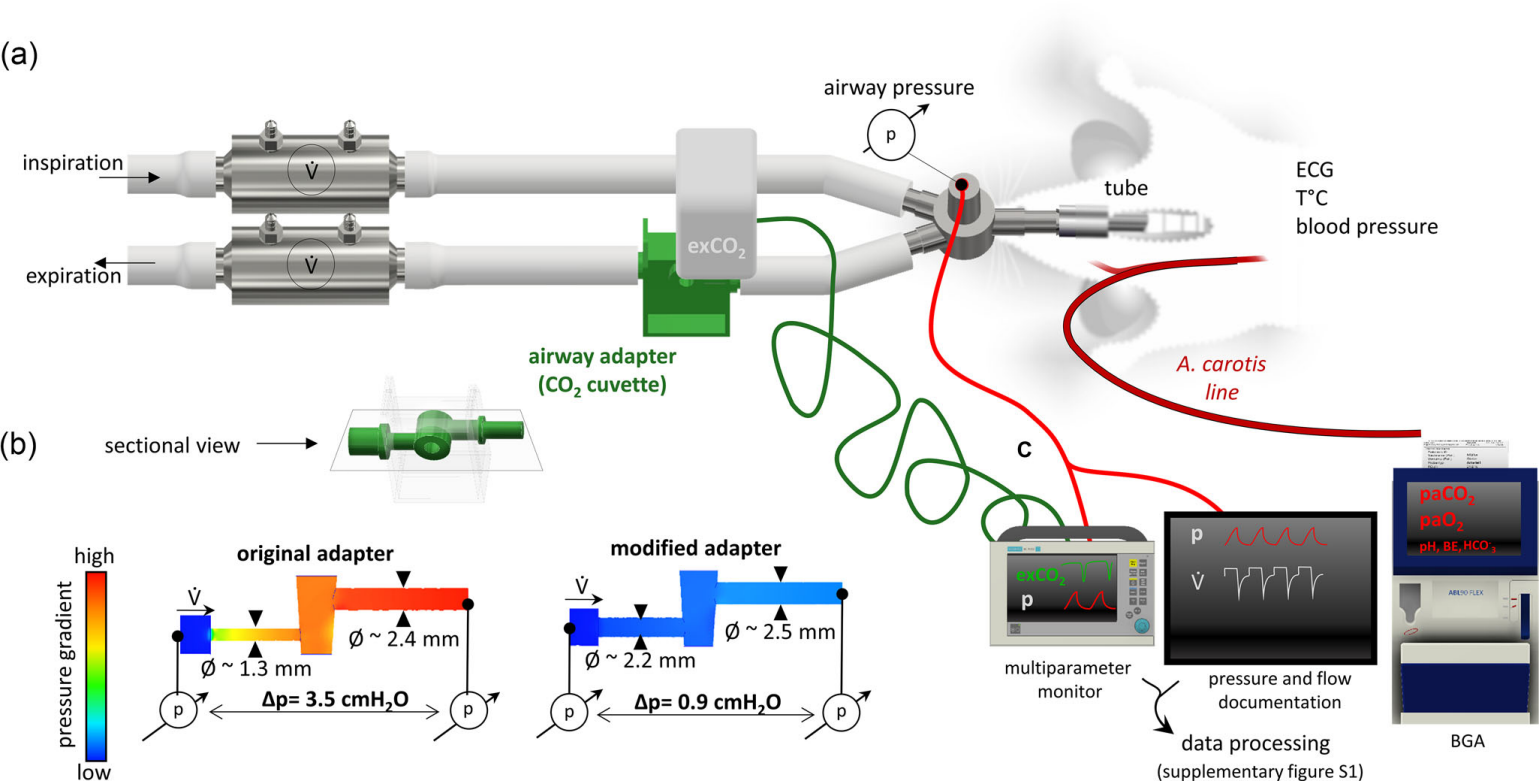
Experimental set‐up. (a) Breathing circuit with flow (*V̇*) and airway pressure (p) measurement of an anaesthetized, tracheotomized and mechanically ventilated rat. The airway adapter (CO_2_ cuvette, green) for continues mainstream measurement of exhaled CO_2_ (exCO_2_) is placed in the expiratory limb. Arteria carotis is catheterized for blood sampling for blood gas analysis (BGA) and blood pressure monitoring. Heart rate (by electrocardiogram; ECG) and temperature were monitored through the entire experiment. (b) Pressure gradient through the original (left) and modified airway adaptor (right) was simulated using (Autodesk CFD v.2024 software) and confirmed by pressure measurements (at flow of 30 mL s^−1^). (c) Synchronized documentation of *V̇*, *P*, exCO_2_ and $P_{\mathrm{aC}O_{2}}$. Airway pressure is documented simultaneously with the exCO_2_ on the multiparameter monitor and with *V̇* on the respiratory data recordings.

A conventional sidestream airway adaptor (CO_2_ cuvette) was inset in the expiratory limb of the breathing circuit for continues mainstream exCO_2_ measurement (Figure 1a). Changes in the diameter of the breathing circuit due to the insertion of additional components (as is the case with the CO_2_ cuvette) are associated with changes in the flow profile (e.g., turbulences). This results in an increase in differential pressure, which may interfere with the flow and pressure measurements. Therefore, the diameter of the tubing connectors of the CO_2_ cuvette were aligned with that of the breathing circuit to reduce differential pressure (Figure 1b; Supporting information Figure S1). The exCO_2_ was displayed and recorded using a Siemens SC7000 multiparameter monitor equipped with an end‐tidal CO_2_ module (capnostat sensor, Dräger Medical, Lübeck, Germany).

### 2.3 Animal experiments

Male Sprague–Dawley rats with a body weight of 378 ± 31 g (Janvier Labs, Saint‐Berthevin, France) were anaesthetized with 100 mg kg^−1^ ketamine and 1 mg kg^−1^ medetomidine intraperitoneal, and placed on a heating pad to keep body temperature in the range between 36 and 37°C. Subsequently, animals were subjected to tracheotomy, followed by arteria carotis catheterization for blood sampling as described (Spassov et al., 2020). All experiments were performed with seven animals per group (*n* = 7). In the first experimental run, to assess construct validity (Hartmann et al., 2023; Thomsen et al., 2025), animals were subjected to volume controlled ventilation (VCV) with inspiratory fraction of oxygen ($F_{iO_{2}}$) of 0.3, ratio of inspiration to expiration (I:E) of 1:2 and *V*_T_ = 8 mL kg^−1^. The respiratory rate (RR) was stepwise changed (45, 50, 55 and 60 min^−1^) in order to achieve $P_{\mathrm{aC}O_{2}}$ in the whole physiological range between 35 and 45 mmHg. In subsequent runs, to assess criterion validity (Hartmann et al., 2023; Thomsen et al., 2025), animals were ventilated using VCV with a *V*_T_ of either 7 or 6 mL kg^−1^. The RR setting was guided by exCO_2_ measurement, with the objective of maintaining $P_{\mathrm{aC}O_{2}}$ between 35 and 45 mmHg, while maintaining proper oxygenation. In the present study, oxygenation was assessed using the Horowitz index (HI, the ratio of the partial pressure of oxygen in arterial blood and the fraction of inspired oxygen, $P_{aO_{2}}$/$F_{iO_{2}}$). HI allows for immediate scoring of lung function and ventilation efficiency. At the end of the experiments, animals were exsanguinated under deep anaesthesia (intraperitoneal application of two times the initial anaesthesia dose, 200 mg kg^−1^ ketamine/2 mg kg^−1^ medetomidine).

### 2.4 Measurement and data collection

To ensure respiratory gas steady state, BGA and exCO_2_ recordings were performed 30 min after the beginning of mechanical ventilation and 30 min after changing respiratory rate. Levels of exCO_2_, airway pressure (*P*), inspiratory and expiratory flows (*V̇*) were recorded in a synchronized manner. In addition, the airway pressure line was split to be recorded simultaneously with the CO_2_ curve on the multiparameter monitor and with the flow curves using DataGrabber (Dräger Medical) and LabView (National Instruments, Austin, TX, USA) software. Body temperature, mean blood pressure and heart rate were monitored continuously through the experiment. The exCO_2_ levels were compared to the $P_{\mathrm{aC}O_{2}}$ levels from the BGA (ABL90 Flex, Radiometer, Germany; Figure 1c). We further analysed $P_{aO_{2}}$, pH, lactate (Lac), base excess (BE) and bicarbonate (HCO_3_^−^).

### 2.5 Statistics

Data are presented as means ± SD or box plots for *n* = 7/group. One‐way ANOVA followed by Tukey's *post hoc* test or Student's paired *t*‐test was used for multiple and pairwise comparison, respectively. A *P *< 0.05 was considered significant. Correlation and normal distribution (Shapiro–Wilk test) were analysed. Agreement between exCO_2_ and $P_{\mathrm{aC}O_{2}}$ was assessed by Bland–Altman analysis (Giavarina, 2015). Corresponding data points are presented as scatterplots or histogram. For all statistical analysis GraphPad Prism (v.10.2.3, GraphPad Software, Boston, MA, USA) was used.

## 3 RESULTS

The validity of the mainstream exCO_2_ measurements, as an estimate of the $P_{\mathrm{aC}O_{2}}$ steady state, was established in rats during mechanical ventilation with different *V*_T_ and respiratory rates. Physiological and experimental conditions, for example, body temperature, heart rate or blood pressure were comparable in all measurements (Table 1).

**TABLE 1:** Experimental and physiological conditions.

*V*_T_	8 mL kg^−1^				7 mL kg^−1^	6 mL kg^−1^
RR (min^−1^)	45	50	55	60	58 ± 1	68 ± 1
*T* (°C)	36.7 ± 1.3	36.6 ± 0.6	36.3 ± 0.6	36.4 ± 0.3	36.5 ± 0.3	36.4 ± 0.3
HR (beats min^−1^)	299 ± 17	312 ± 25	308 ± 32	325 ± 18	302 ± 18	314 ± 27
mBP (mmHg)	94 ± 14	115 ± 16	108 ± 6	102 ± 13	100 ± 13	99 ± 12
pH	7.430 ± 0.371	7.453 ± 0.290	7.489 ± 0.037*	7.488 ± 0.040	7.499 ± 0.041	7.463 ± 0.019
Lac (mmol L^−1^)	0.7 ± 0.2	0.7 ± 0.2	0.7 ± 0.2	0.6 ± 0.1	0.9 ± 0.2	1.1 ± 0.3
BE (mmol L^−1^)	6 ± 2	5 ± 2	5 ± 2	4 ± 2	4 ± 2	6 ± 2
HCO_3_^−^ (mmol L^−1^)	29 ± 2	29 ± 2	29 ± 2	28 ± 2	28 ± 2	29 ± 1

*Note*: ANOVA followed by Tukey's *post hoc* test, *P = 0.0137 versus RR of 45.

Abbreviations: BE, base excess; HCO_3_^−^, bicarbonate; HR, heart rate; Lac, lactate; mBP, mean blood pressure; RR, respiratory rate.

**FIGURE 2:**
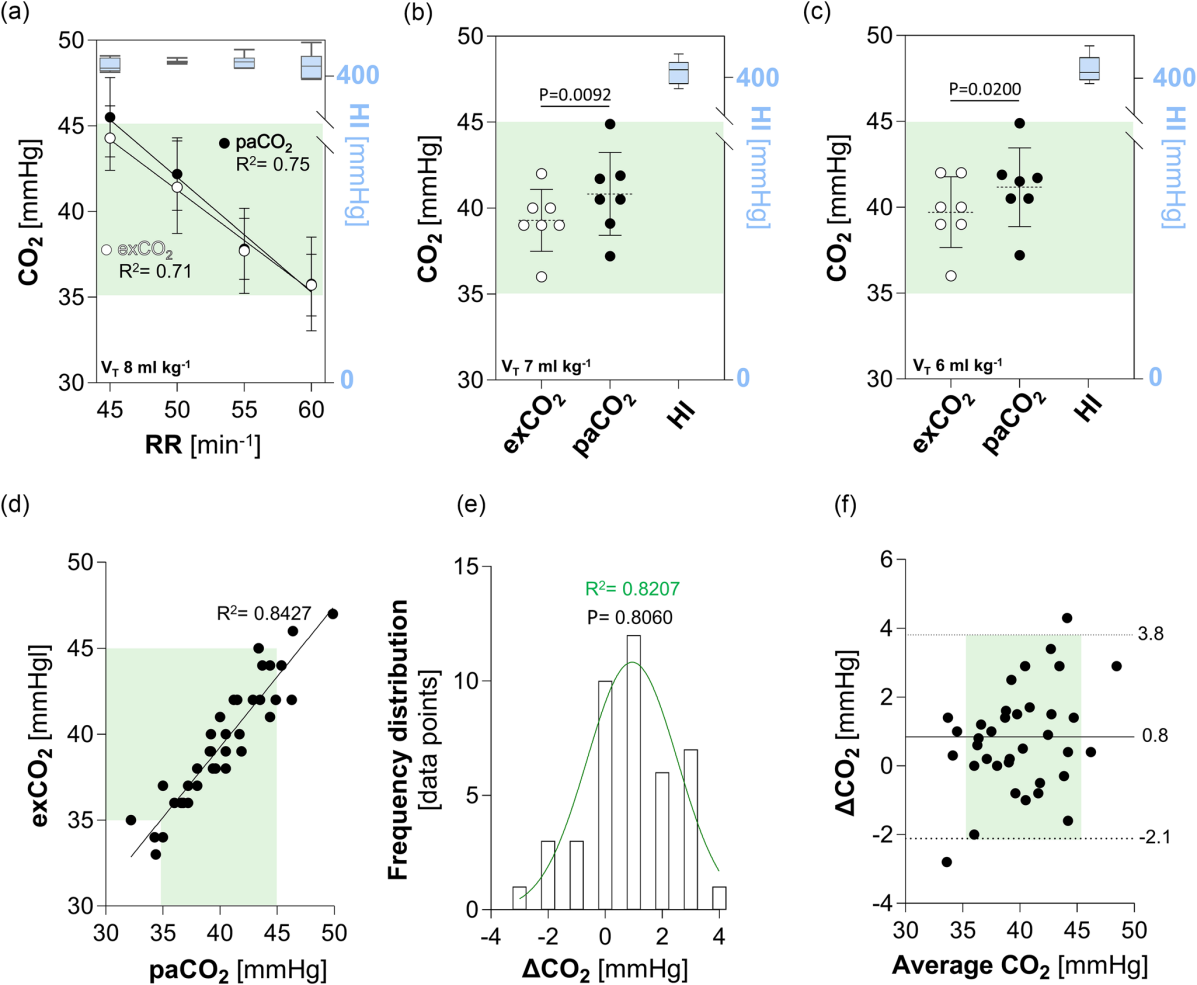
Measurements of exCO_2_ and $P_{\mathrm{aC}O_{2}}$. (a) CO_2_ levels measured in exhaled breath (exCO_2_) and BGA ($P_{\mathrm{aC}O_{2}}$) at *V*_T_ 8 mL kg^−1^ and oxygenation (Horowitz Index (HI), blue) in response to respiratory rate (RR). Green shading indicates the physiological range of CO_2_. Regression analysis of exCO_2_ and $P_{\mathrm{aC}O_{2}}$ depending on respiratory rate. All *n* = 7/group. (b, c) exCO_2_ and $P_{\mathrm{aC}O_{2}}$ levels at *V*_T_ of 7 mL kg^−1^ (b) and 6 mL kg^−1^ (c) with respiratory rate set to maintain 40 mmHg in exhaled breath, while preserving proper oxygenation (HI, blue). All *n* = 7/group. *t*‐test. (d) Correlation between exCO_2_ and $P_{\mathrm{aC}O_{2}}$ measurements. Regression analysis included all 42 CO_2_ data points. (e) Normal distribution of the difference between the two measurement methods ∆CO_2_ = ($P_{\mathrm{aC}O_{2}}$ − exCO_2_). Histogram, Gaussian fit and Shapiro–Wilk test for normal distribution (α = 0.05) including all 42 CO_2_ measurements. (f) Bland–Altman mean‐difference plot (mean CO_2_ = ($P_{\mathrm{aC}O_{2}}$ + exCO_2_)/2) with bias (continuous line) and limits of agreement (dotted lines) including all 42 CO_2_ data points.

Mechanical ventilation with *V*_T_ = 8 mL kg^−1^ resulted in respiratory rate‐dependent changes within the physiological range (35 and 45 mmHg) equally mirrored in both $P_{\mathrm{aC}O_{2}}$ and exCO_2_ (Figure 2a, green shade), while maintaining uniform sufficient oxygenation. Change of RR from 45 to 55 min^−1^ was associated with a variation in pH in the range 7.430 to 7.489, respectively (*P* = 0.0137; Table 1). RR did not affect lactate, base excess or bicarbonate levels (Table 1).

During mechanical ventilation with *V*_T_ = 7 or 6 mL kg^−1^, exCO_2_ was on average 1.5 mmHg lower compared to $P_{\mathrm{aC}O_{2}}$ (*P* = 0.0092 and *P* = 0.02, respectively; Figure 2b,c).

All exCO_2_ measurements correlated with $P_{\mathrm{aC}O_{2}}$ (*P *< 0.0001), also beyond the physiological CO_2_ range (Figure 2d). The differences between the levels of measured CO_2_ in exhaled breath and by BGA showed a normal distribution (Figure 2e).

A Bland–Altman plot showed CO_2_ mean/difference data points virtually all randomly scattered around the difference mean, and within the standard deviation limits (Figure 2f).

## 4 DISCUSSION

Mechanical ventilation is necessary in pharmacological and physiological studies, particularly where precise respiratory control is crucial to the study's outcomes and reproducibility. In this regard a strict control of the steady state of respiratory gases is an essential experimental condition. However, there are no ‘universal’ ventilation settings that guarantee a certain respiratory gas balance. In fact, the respiratory efficiency depends on the ventilator settings (e.g., *V*_T_, RR) but it will vary depending on the ventilator performance, the external dead space (e.g., tubing, Y‐piece) and on the used animal model (e.g., species and health state).

The approach presented for continuous monitoring of exhaled CO_2_ in small animals uses a modified CO_2_ cuvette for mainstream measurement. This allows accurate CO_2_ detection by avoiding an increase of dead space. By adjusting the diameter of the CO_2_ cuvette connectors to the diameter of the airway tubing, pressure differences and the associated influence on the respiratory measurements were reduced (Supporting information Figure S1), ensuring the reliability of the documented respiratory mechanics data. In addition, the approach is non‐invasive, which makes it applicable to a wide range of models. This new measure can be applied without interfering with the specific experimental set‐up, while contributing to the refinement of animal experiments and reproducibility of research results.

In the present study, mechanical ventilation with a *V*_T_ = 6–8 mL kg^−1^ was adequate to maintain efficient gas exchange in medium‐sized small animals, that is, rats. In particular, the animals were adequately oxygenated at all tidal volumes or respiratory rates. Blood pH, base excess and bicarbonate levels appeared to be at their upper physiological limit for anaesthetized rats (Subramanian et al., 2013). The slight shift in the blood acid–base balance in the present model is at least partly related to respiration, for example, to the decrease in $P_{\mathrm{aC}O_{2}}$ with mechanical ventilation at higher respiratory rates. Nevertheless, mild alkalosis may occur in rats due to metabolic, adaptive or compensatory responses (Hopkins et al., 2025; Pepelko & Dixon, 1975; Sur & Hashmi, 2024). Since pH, $P_{\mathrm{aC}O_{2}}$ and exCO_2_ are measured independently, it is unlikely that changes in pH may have directly interfered with the CO_2_ detection. However, CO_2_ is intimately involved in the blood acid–base balance. Therefore, in response to alkalosis, CO_2_ would be preferably ‘retained’ in blood, which may result in reduced CO_2_ in the exhaled breath. Considering $P_{\mathrm{aC}O_{2}}$ levels and the bloods buffer capacity (BE and HCO_3_^−^) such a compensatory mechanism is not obvious. In addition, in lungs of healthy individuals, the end‐tidal CO_2_ is typically about 2–5 mmHg higher than $P_{\mathrm{aC}O_{2}}$ due to external, anatomical and physiological dead space (Long et al., 2017; Thompson & Jaffe, 2005). In the present study, exCO_2_ was randomly higher or lower than the corresponding $P_{\mathrm{aC}O_{2}}$ values. The exact reasons for the atypical variances between exCO_2_ and $P_{\mathrm{aC}O_{2}}$ may be rationalized by the positioning of the CO_2_ cuvette. This may by associated with inhomogeneous mixing of inspiratory and expiratory air during expiration, and/or by continued CO_2_ diffusion in the expiratory limb of the breathing circuit during inspiration. It may be further speculated that technical issues, such as the response time of the sensor and the sampling rate of the monitor, or gas exchange that may occur in the small volumes of sampled blood prior to BGA, may also contribute to this irregularity.

Nevertheless, the presented mainstream approach of CO_2_ quantification showed a high degree of agreement with the standard measurement (correlation, Bland–Altman analysis). The high accuracy between exCO_2_ and $P_{\mathrm{aC}O_{2}}$ suggests that non‐invasive CO_2_ measurements in the expiratory limb are feasible in smaller animals during mechanical ventilation without adding additional dead space. The statistical evidence is fully consistent with the physiological limits and allows to suggest that the exCO_2_ is a valid proxy for continuous monitoring and control of $P_{\mathrm{aC}O_{2}}$ during mechanical ventilation. However, the applicability of this finding to smaller animals (e.g., mice) requires further investigation.

This new approach may not only be of interest in the frame of science but may also be implemented easily in the veterinary practice routine. Although, not commonly used, mechanical ventilation is an essential tool to ensure safety and stability during surgery and/or in critically ill animal patients (Ambrosio & Fantoni, 2024), which are faced with the side effects of invasive respiratory efficiency monitoring.

The measurements of both exCO_2_ and $P_{\mathrm{aC}O_{2}}$ demonstrated agreement, even at values that fell outside the normocapnic range, for example, at $P_{\mathrm{aC}O_{2}}$ of 33 or 50 mmHg. On this basis and considering the linearity of CO_2_ within in the normocapnic range, it is presumed that the exCO_2_ measurements will retain their validity across a broader CO_2_ range. However, the applicability of the exCO_2_ measurement during hypo‐ or hyperventilation has to be addressed in future studies. Beyond the detection of exhaled CO_2_, the approach presented here may have some potential to be used for the analysis of the CO_2_ dynamics (capnometry) during the respiratory cycle (Long et al., 2017; Ward & Yealy, 1998). Indeed, synchronized documentation of exCO_2_ and ventilation–airflow curves may be used to calculate the amount of exhaled CO_2_ during the respiratory cycle (Supporting information Figure S2). It has been suggested that changes in the effectively transported CO_2_ volume may be useful in assessing disease severity, efficiency of mechanical ventilation and ventilation–perfusion ratio. However, its diagnostic value and potential to guide ventilation therapy remain to be proven (Verscheure et al., 2016).

### 4.1 Conclusion

The presented non‐invasive measurement could be used to assess CO_2_ in the expiratory air of small animals continuously, without compromising ventilation efficiency due to additional death space. The exCO_2_ mirrors $P_{\mathrm{aC}O_{2}}$ precisely and therefore can reduce the number of blood gas analysis. The continuous monitoring of exCO_2_ can be used to maintain $P_{\mathrm{aC}O_{2}}$ in the physiological range during general anaesthesia with mechanical ventilation.

## AUTHOR CONTRIBUTIONS

Christin Wenzel, Bernd Flamm, Stefan Schumann: conception and design of the study. Christin Wenzel, Stefan Schumann: performed experiments. Christin Wenzel, Silke Borgmann, Bernd Flamm, Lea Kuhn, Stefan Schumann, Johannes Schmidt, Johannes Spaeth, Sashko Spassov: acquisition analysis and interpretation of data. Christin Wenzel, Sashko Spassov: manuscript drafting. All authors have revised and approved the final version of the manuscript, agreed to be accountable for all aspects of the work in ensuring that questions related to the accuracy or integrity of any part of the work are appropriately investigated and resolved, and all persons designated as authors qualify for authorship, and all those who qualify for authorship are listed.

## CONFLICT OF INTEREST

None declared.

## Supporting information



Figure S1. Effects of the cuvette modification on respiratory data.

Figure S2. Determination of total CO_2_ in the exhaled breath.
_2_

## Data Availability

All data relevant to the manuscript are shown in the main text or as supplements. All raw data that support the findings of the study are available upon reasonable request.
