# Supplementary material for: Continuous carbon dioxide monitoring in the exhaled breath of mechanically ventilated rats
Source: Exp Physiol. 2025 Sep 14;111(3):614–9. doi: 10.1113/EP093058 (PMC12949111; doi:10.1113/EP093058)
Supplement: Supplementary file 1 — Figure S1. Effects of the cuvette modification on respiratory data. [file EPH-111-614-s001.pdf]

Supporting materials

A

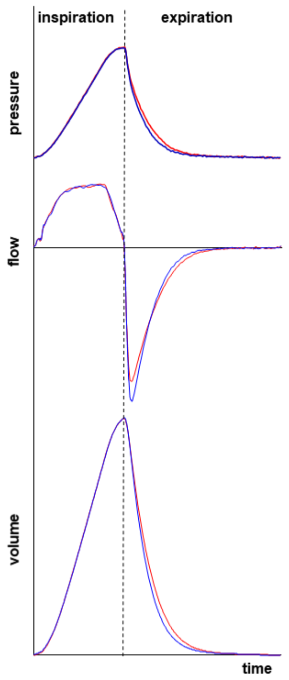

B

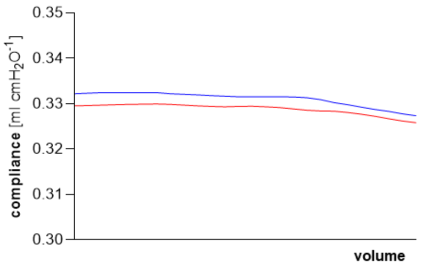

C

Impact of the cuvette concertos diameter on respiratory data.

parameter	original	modified	Δ [%]
P_{max} [cmH20]	14.53	14.52	0.07
P_{mean} [cmH20]	8.59	8.49	1.18
$I\dot{V}_{max}$ [ml s^{-1}]	9.17	9.17	0.00
$E\dot{V}_{max}$ [ml s^{-1}]	19.77	26.63	12.64
V_{max} [ml]	2.58	2.59	0.39
V_{mean} [ml]	0.69	0.66	4.55
compliance*	0.329	0.331	0.682

P= pressure, $I\dot{V}$= Inspiratory flow, $E\dot{V}$= expiratory flow,
V= volume Δ= absolute differences between original and modified cuvette

Supplementary figure S1. Effects of the cuvette modification on respiratory data. A) Pressure, flow and volume curves for one breath recorded during mechanical ventilation with the original (red) or the modified (blue) CO_2 cuvette. B) Corresponding dynamic compliance; *calculated using the gliding-SLICE method (Schumann et.al, Physiol. Meas. 2009, 30, 1341-1356) and C) respiratory parameters.

The effect of matching the internal diameter of the cuvette connectors to the size of the tubing was assessed using the setup shown in the main manuscript (fig. 1) using a lung model instead of the animal. The original and the modified cuvettes were placed in the same position in the expiratory limb and with identical ventilator settings. The modified cuvette showed a lower differential pressure (fig. 1 in the main manuscript) and a small delay of the pressure decay and slightly reduced peak flow during expiration, correspondingly the expiratory course volume decline was also delayed (fig. S1A). The modified cuvette showed slightly better compliance (fig. S1B). Peak pressure (P_{max}), peak inspiratory flow ($I\dot{V}_{max}$) and tidal volume (V_{max}) were comparable (fig. S2C). However, the mean pressure over time (P_{mean}) peak expiratory flow ($E\dot{V}_{max}$) and mean tidal volume over time (V_{mean}) were considerably different between the original and the modified cuvettes.
