# Supplementary material for: Continuous carbon dioxide monitoring in the exhaled breath of mechanically ventilated rats
Source: Exp Physiol. 2025 Sep 14;111(3):614–9. doi: 10.1113/EP093058 (PMC12949111; doi:10.1113/EP093058)
Supplement: Supplementary file 2 — Figure S2. Determination of total CO2 in the exhaled breath. 2 [file EPH-111-614-s002.pdf]

Supporting materials

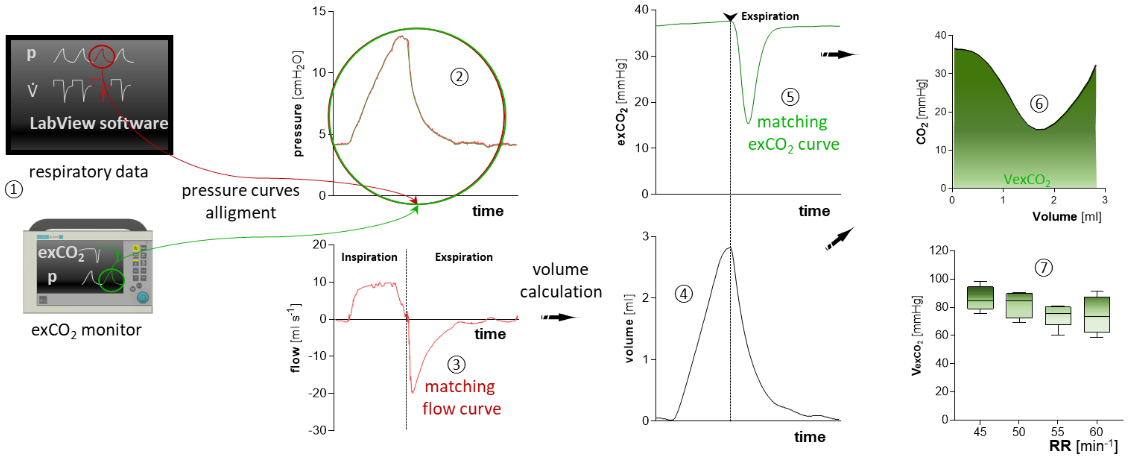

Supplementary figure S2. Determination of total CO_2 in the exhaled breath. ① Airway pressure line was split to be recorded simultaneously with the airway flow and $exCO_2$ using LabView (National Instruments Corporation, TX, US) and the Siemens SC 7000 Monitor, respectively (fig. 1 in the main manuscript). ② Matlab software (MathWorks Inc., CA, US) was used for airway pressure curves alignment needed to identify i) the matching airway flow ③ for calculation of the applied tidal volume ④ and ii) the matching $exCO_2$ curve ⑤. The area under the curve (green shaded area) was calculated as quantity of the actual volume of expired CO_2 (V_{exCO2}) ⑥, ⑦ Relation between total $exCO_2$ and respiratory rate.

Simultaneous documentation of the airway pressure in the respiratory and CO_2 documentation systems allowed matching the airway flow/tidal volume curves with the corresponding $exCO_2$ curve. The $exCO_2$ is measured in the expiratory limb of the breathing circuit. Therefore it does not show the typical features of end-tidal CO_2 curve (i.e. baseline=0 mmHg, expiratory upstroke, alveolar plateau and inspiratory down stroke), which are usually used for capnometry analysis. Furthermore, the $exCO_2$ curve may be affected by inhomogeneous mixing of inspiratory and expiratory air and diffusion of CO_2 during inspiration or zero flow phases or reflects the response time of the capnostat sensor. Correspondingly, the CO_2 time course with increasing exhaled volume (used to calculate the area under the curve as an estimate of the actually eliminated CO_2 (‘area X’, Verscheure, S., et al., Crit Care, 2016. **20**(1): p. 184)) is also untypical. Nevertheless, even with untypical time course, the area under the curve of detected CO_2 during expiration correlated with the amount of effectively transported CO_2 per respiratory cycle. Indeed, the levels of actually eliminated CO_2 showed clear respiratory rate dependency, implicating, that the presented approach may provide an additional means for estimation of the mechanical ventilation efficiency. However, the potential of the mainstream exhaled CO_2 dynamics to be used for calculation of dead space or to indicate changes in ventilation-perfusion relationships or disease state during mechanical ventilation is to be established and validated in the future.
